# Women’s experiences of safety apps for sexualized violence: a narrative scoping review

**DOI:** 10.1186/s12889-021-12292-5

**Published:** 2021-12-30

**Authors:** Nicole Doria, Christine Ausman, Susan Wilson, Annalisa Consalvo, Jad Sinno, Leah Boulos, Matthew Numer

**Affiliations:** 1 Dalhousie University, 6299 South St, Halifax, NS B3H 4R2 USA; 2 University of Toronto, 155 College St Room 500, Toronto, ON M5T 3M7 USA; 3 Maritime SPOR SUPPORT Unit, 5790 University Avenue, Halifax, NS B3H 1V7 USA

**Keywords:** Sexualized violence, Safety apps, Scoping review

## Abstract

**Background:**

Sexualized violence against women is a significant human rights problem worldwide. Safety apps have the capacity to provide women with resources to prevent or respond to experiences of sexualized violence.

**Methods:**

The aim of the following study was to review the scope of the literature on women’s experiences of safety apps related to sexualized violence. The databases Embase, MEDLINE, PsycINFO, and Scopus were systematically searched, and seven studies were included in this review.

**Results:**

Thematic analysis identified the following themes in the literature: (1) security; (2) accessibility; and (3) knowledge.

**Conclusion:**

The gaps in the literature are identified and implications and recommendations for future research is discussed.

**Supplementary Information:**

The online version contains supplementary material available at 10.1186/s12889-021-12292-5.

## Background

The high rate of sexualized violence against women is an urgent public health issue and a significant human rights problem [9]. Although this is well known, global prevalence of sexualized violence is difficult to determine as there is not a universal understanding or measurement of what constitutes sexualized violence [36]. Further, only an estimated 32-35% of incidents of sexualized violence in the United States are reported to police [66, 67]. In 2018, 652,676 women reported being raped or sexually assaulted nation-wide in the United States [62]. Canadian data suggests even lower reporting rates of sexual assault, with only 5% reported to police [23, 24]. Self-reported data from the 2014 General Social Survey (GSS) on Canadians’ Safety (Victimization) shows that there were 22 incidents of sexual assault for every 1,000 Canadians aged 15 and older, which represented approximately 636,000 incidents of sexual assault [23]. In the United Kingdom, the Crime Survey for England and Wales estimated that 20% of women experience some type of sexual assault after the age of 16, which is equivalent to 3.4 million female victims [49]. Sexualized violence, however, is a broad term that encompasses much more than sexual assault and rape. Sexualized violence includes any unwanted sexual contact that targets sexuality and is physical and/or psychological in nature, including but not limited to sexual abuse, sexual assault, rape, sexual harassment, stalking, indecent/sexualized exposure, degrading sexual imagery, sharing sexual photographs without permission, and/or unwanted comments or jokes [71, 76]. Although anyone can experience sexualized violence, women are primarily the victims and are most likely experience more severe forms of violence, whereas men are typically the perpetrators [14, 65]. Globally, one in three women experience physical and/or sexualized violence in their lifetime, mostly perpetrated by a current or former intimate partner, and six percent of women experience sexualized violence from someone other than an intimate partner [69, 76].

### Marginalized Populations

According to the Canadian census, individuals who are young, female, Indigenous, and/or a gender or sexual minority are at greater risk for experiencing sexual assault than the general population [23]. Similar trends are noted in the United States, where a study from Colorado revealed that bisexual women are more than twice as likely to experience sexualized violence compared to straight identifying women [43]. The authors also found that 26% of transgendered participants have experienced sexualized violence and that their gender identity was the motivating factor for the assault [43]. Further, a 2018 review of the literature on technology-facilitated sexualized violence reported that women, as well as, lesbian, gay, bi-sexual, trans, and intersex individuals are at an increased risk of being the targets of online abuse [38]. These findings suggest that members of the sexual and gender diverse community are at a higher risk for experiencing sexualized violence, however, further research is needed in this area [38, 48].

### Health Outcomes and Traditional Resources

The negative health outcomes associated with physical sexual assault are well known and include poorer self-rated health, exacerbated psychological distress, and post-traumatic stress disorder (PTSD) [3, 19, 41, 53, 61, 78]. Literature that focuses on the health outcomes associated with other forms of sexualized violence, however, is scarce [50, 56, 63]. A recent Australian study found that women’s experiences of sexualized violence were most commonly public harassment, flashing, unwanted groping, or being coerced into consenting to sex [65]. These women were at an elevated risk for having anxiety, depression, and PTSD as compared to women who did not experience sexualized violence [65]. Another study reported that one-third of women who experienced sexualized violence were later diagnosed with PTSD [47]. Negative mental health outcomes have been found to interfere with the victim’s physical functioning and interpersonal relationships [7, 8, 31, 68].

A variety of prevention initiatives, supports, and resources exist that aim to increase safety for women, reduce incidents of sexualized violence, and help offset the worsened health outcomes. Some examples include, educational campaigns, medico-legal services, specific training for healthcare professionals, prophylaxis for HIV infection, and centres that provide trauma-informed care [75]. Unfortunately, these initiatives and resources are often limited to high-income and resource-rich settings [76]. Further, as sexualized violence continues to rise world-wide, there is reason to believe that the current measures to address sexualized violence are insufficient (UN Women, 2019). In the majority of countries with available data, less than 40% of women who experienced violence sought help of any sort [70]. Among women who did seek help, most reached out to family and friends instead of formal institutions, and less than 10% of women sought help from the police [70]. These statistics demonstrate a need for safe, accessible, affordable, and comprehensive resources that aim to prevent sexualized violence against women and support women who have experienced sexualized violence.

### Mobile Technology and Safety Apps

In today’s digital age, mobile technology presents a unique opportunity to deliver improved sexualized violence interventions and resources [34]. Research shows that 95% of adults between the ages of 18 and 34 in the United States own a mobile phone [60]. Further, mobile technologies as sophisticated as smartphones are widely used around the world: 95% in South Korea, 88% in Israel, 81% in Australia, 80% in Spain, with a median worldwide ownership of smartphone technology of 76% [59]. The extant literature has primarily focused on the ways that mobile apps can be used for health and fitness [1, 15, 16]. There has recently, however, been interest in discovering the potential of using smartphone technology to help enhance women’s safety.

A recent review on the role of emerging technologies regarding women’s safety found that smartphones were the most commonly discussed protective technology in the literature (37.2% mobile phones, 18.8% apps) [20]. Smartphones can be used to call for help in emergencies, film violence incidents/injuries, retain threatening text messages, and use apps to contact support networks [20]. Another study found that 62.9% of college students would consider downloading a personal safety app, and even more students would consider doing so if the app contained a tracking feature [46]. Women were also found to be more likely than men to download a safety app with tracking features to increase their sense of security and reassurance [46]. Overall, the proliferation of technological solutions for sexualized violence such as signal-/alarm-emitting wearables and apps have surged in recent years [74].

Specific to sexualized violence, qualitative evidence shows that technology can provide access to information and services, strengthen support networks, and support sexualized violence victims in their safety and escape planning [77]. An Australian review found that safety apps for public stranger violence focused mainly on location-based services, personal alarms, and crowd-sourced data [45]. This review identified *MySafetyPin*, *Saven*, and *My Keeper* as useful safety apps allowing users to identify dangerous areas, and the *Women Safety Totem SOS* app for providing information to help reduce the likelihood of being a target for violence and tips on how to handle violence (e.g., self-defence information) [45]. Although there are several studies that have explored safety apps regarding sexualized violence, a preliminary search for existing reviews and protocols in MEDLINE, PROSPERO, and Open Science Framework in January 2020 revealed that the findings regarding women’s experiences and perceptions of these apps has not been comprehensively reviewed. Therefore, the research question for this scoping review was: What is the scope of the literature on women’s experiences of safety apps in relation to sexualized violence?

## Method

A scoping review method was chosen to comprehensively review the published literature on women’s experiences using safety apps related to sexualized violence [5]. The aim was to map the extant literature and research gaps on the experiences of women who use sexualized violence related safety apps, including the barriers of using such apps [5]. This scoping review employed a systematic methodology guided by the Arksey and O’Malley [5] framework. This framework includes identifying the research question, identifying relevant studies, selecting relevant studies, charting the data, and collating and summarizing the findings [5]. Critical appraisal was not conducted, as the purpose of a scoping review is to provide an overall picture of a body of evidence on a topic, and not to assess quality and rigour [51].

### Identifying Relevant Studies

The literature search for this scoping review was conducted in January 2020. The following electronic databases were searched: MEDLINE All (Ovid), Embase (Elsevier Embase.com), PsycINFO (EBSCOhost), and Scopus (Elsevier Scopus.com). Our search strategy was developed in consultation with a medical librarian at the Maritime SPOR SUPPORT Unit (LB). The search strategy was designed in MEDLINE All and Scopus between January 21 and 23, 2020, and tested using a small set of relevant articles previously identified by the review team [12, 18, 33, 74]. Once finalized, the MEDLINE All and Scopus searches were then translated to the other databases (Embase and PsycINFO) according to the controlled vocabulary and search syntax requirements of each database. No language limits or other published search filters were applied, but an *ad hoc* filter was developed to limit studies to those related to women. The MEDLINE All search strategy can be seen in Table 1, and translations of the search to all other databases can be found in Appendix A. Grey literature searching was not conducted separately, but any grey literature indexed in the databases searched (e.g., conference proceedings in Embase and Scopus) were not excluded from the search results. All searches were executed and results exported on January 23, 2020. References were deduplicated in EndNote X9 by the medical librarian according to the method developed by Bramer et al. [13].

**Table 1:** Ovid MEDLINE All search strategy, subsequently translated to other databases and executed on January 23, 2020

1	exp Intimate Partner Violence/
2	exp Sex Offenses/
3	(anti-abuse or anti-assault or anti-harassment or anti-rape or anti-victim* or anti-violence).ti,ab,kw,kf.
4	((dating or domestic or gender* or partner* or relationship* or wom#n or sex*) adj2 (abuse* or assault* or violence)).ti,ab,kw,kf.
5	(intimate partner violence or ipv).ti,ab,kw,kf.
6	(rape or rapes or raped or rapist* or raping).ti,ab,kw,kf.
7	(sex* adj2 coerc*).ti,ab,kw,kf.
8	(sex* adj2 (force* or unwanted or unwelcome)).ti,ab,kw,kf.
9	(sex* adj2 harass*).ti,ab,kw,kf.
10	(sex* adj2 victimi*).ti,ab,kw,kf.
11	(unwanted pursuit or unwanted online pursuit).ti,ab,kw,kf.
12	(wom#n adj4 (safety or security)).ti,ab,kw,kf.
13	or/1-12
14	Mobile Applications/
15	(app or apps).ti,ab,kw,kf.
16	(application* adj4 (android or cell* or iphone* or mobile or smart phone* or smartphone*)).ti,ab,kw,kf.
17	or/14-16
18	13 and 17

Reference lists of systematic reviews, scoping reviews, and literature reviews that were found through the search of databases were also checked to ensure all relevant studies had been screened – including reference lists of the included studies. Last, four key journals were identified for hand-searching (Journal of Technology in Human Services, Violence Against Women, BMC Public Health, and Health Promotion International). The table of contents for the last five years (2015-2020) were searched for each journal to identify any articles that may have been missed in the database search.

### Selecting Relevant Studies

All of the research articles from the database search were imported into Covidence (an online software tool for review management) for organization and screening. Inclusion and exclusion criteria were established for selecting relevant studies prior to screening began (Table 2). Our definition of sexualized violence used was broad to include any unwanted sexual contact that targets sexuality and is physical and/or psychological in nature, including sexual abuse, sexual assault, rape, sexual harassment, stalking, indecent/sexualized exposure, degrading sexual imagery, sharing sexual photographs without permission, and/or unwanted comments or jokes [71, 76]. Sexualized violence experienced or perpetrated by a stranger or by a current or former dating/intimate partner was included. We considered a sexualized violence-based safety app for women to be any app that protected women from danger, risk, or injury related to past, present, or future sexualized violence, including outcomes related to emotional, physical, psychological, and/or sociological health. We sought research studies that collected primary qualitative data as we were only interested in identifying women’s experiences for the purposes of this scoping review, which cannot be well captured by quantitative data [29]. Literature reviews, systematic reviews, conference proceedings, and literature that did not include empirical data (commentaries, editorials, book reviews) were excluded. Mixed-methods studies were reviewed, however, only the qualitative components were considered and included if relevant. Sexualized violence interventions and resources that are meant for children or teenage girls are typically designed to meet the unique needs of minors, which differ from the needs of adults [37]. Given that our research question was interested in understanding the experiences of adult women, inclusion criteria was limited to 18 years of age or older; if an age range was not included in the study or the age range included any participants that were 17 years of age or younger, the study was excluded. Studies that had male participants were included as long as the data on women could be extracted. Studies conducted worldwide that were published in English or French were also included and all publication dates were considered for inclusion.

**Table 2:** Inclusion and Exclusion Criteria for Selected Articles

Inclusion Criteria	Exclusion Criteria
• Research articles including primary research. • Safety apps related to sexualized violence: any unwanted sexual contact that targets sexuality and is physical and/or psychological in nature perpetrated by a stranger or current or former intimate/dating partner • Qualitative findings (including qualitative components of mixed methods studies). • Published in English or French. • Focused on adult women’s (>18 y/o) experiences of sexualized violence focused safety apps. • All dates and all countries.	• Research articles that did not include primary research. • Safety apps not related to sexualized violence. • Quantitative findings. • Not published in English or French. • Data that did not focus on adult women’s (<18 y/o) experiences (only men or not able to extract data on women). • Literature that did not include empirical data (commentaries, editorials, book reviews). • Literature/systematic reviews. • Conference proceedings and dissertations.

Four reviewers (AC, CA, ND, SW) independently screened all articles at the title/abstract stage and full-text stage in accordance with the inclusion/exclusion criteria (each study in Covidence requires the vote of two reviewers). If voting conflicts could not be resolved by the original two reviewers, a final decision was made in collaboration with the full team. If relevance of a study could not be determined at the title/abstract stage, it was voted forward to be reviewed at the full-text stage. Included full-text articles were obtained through online access, Dalhousie University library services, or Dalhousie University document delivery service. If full-text articles could not be obtained through these means, they were excluded. The same process was followed for hand searching.

### Data Charting, Extraction and Synthesis

The following data was extracted and charted from each of the included studies by ND: author/year, title, country, purpose, participants, research method, sexualized violence focus, safety app, key findings. To ensure rigour and accuracy, a second reviewer (CA) reviewed and confirmed all extracted data. Thematic analysis guided by Braun and Clarke [17] was employed to identify emerging themes, which is a method for “identifying, analyzing, and interpreting patterns of meaning (‘themes’) within qualitative data” ([22], p. 297). Each reviewer (AC, CA, ND, SW) familiarized themselves with the included studies, generated initial codes, and searched for themes. Coding and preliminary themes were compared, reviewed, and defined by all reviewers. Inductive (data-driven) thematic analysis was used, which allowed the actual data (included studies) to derive analysis and the themes that emerged [17]. No software was used to organize and analyze the research data.

## Results

**Fig. 1:**
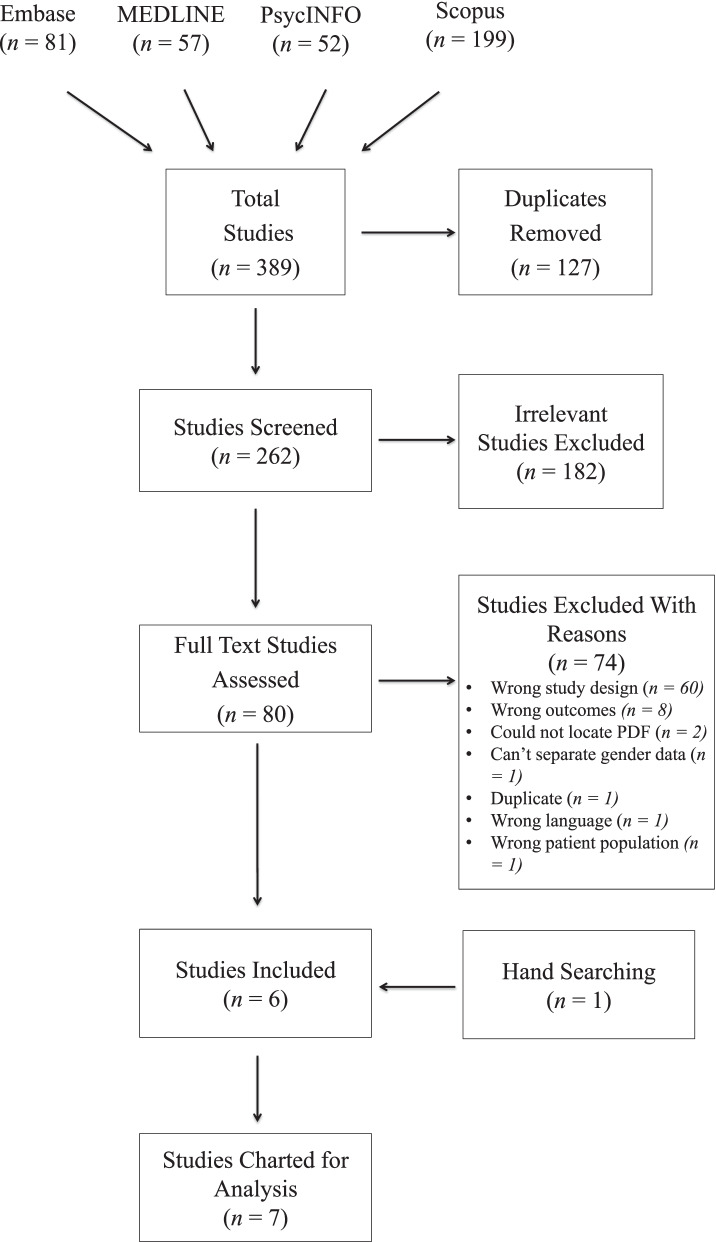
PRISMA flowchart of study selection process

Across the four databases searched, a total of 389 studies were identified; 127 duplicates were removed, resulting in 262 studies screened. Screening at the title and abstract level resulted in the exclusion of 182 studies. There were 80 studies screened at the full-text stage, with 74 studies being excluded – the majority (60) for wrong study design. In total, 6 studies were included from the search of databases and 1 study was included from hand-searching, for a total of 7 included studies (see Fig. 1 for PRISMA diagram). Reference lists of systematic reviews, scoping reviews, and literature reviews that were found through the search of databases were also checked to ensure all relevant studies had been screened. This process did not identify any new studies.

Table 3 provides a summary of the included studies and Table 4 provides a summary of the apps featured in the included studies. Of the seven studies included, four were qualitative [2, 11, 44, 64], two were mixed-methods [10, 32], and one was a formative evaluation [52]. However, only qualitative results from all studies were charted, extracted, and synthesized. All studies used interviews or focus groups to collect qualitative data. The majority (6) of included studies were conducted in the United States [2, 10, 11, 32, 44, 52], with only one study conducted in Australia [64]. All included studies were published in 2013 or later. Most of the included studies’ samples (4 studies) were comprised of college women [2, 10, 11, 44]. The remaining three studies recruited any women who self-reported experiencing sexualized violence [32, 52, 64]. The Gilmore et al. [32] study was the only study to report one participant who identified as neither female nor male. The Blayney et al. [10] study was the only study to report the sexual orientation of participants; the sample was comprised almost entirely of heterosexual women. Although Lindsay et al. [44] did not disclose the sexual orientation of participants, they did report that nearly 16% of their sample, which was entirely female, were in past relationships with abusive females. Five studies reported the ethno-racial makeup of their participants, and all of these studies were comprised of a majority Caucasian/White sample [2, 10, 11, 32, 44]. The included studies explored a variety of apps and focused on different aspects of sexualized violence: three studies focused on dating violence [2, 11, 44], with one study specifically focusing on same-sex dating violence [11]; one study focused on sexual victimization [10]; one study focused on sexual assault [32]; and three studies focused on intimate partner violence [44, 52, 64].

**Table 3:** Summary of Included Studies

Author, Year	Title	Country	Purpose	Participants	Research Method	Sexualized Violence Focus	Safety App	Key Findings
[2]	Development of the MyPlan safety decision app with friends of college women in abusive dating relationships	United States	To explore the perceptions of friends of dating violence survivors regarding the benefits of a safety decision aid, deployed through a smart phone application prototype, for friends of female survivors of dating violence.	Thirty-one college students who self-reported having a friend who had experienced dating violence while in college. Participants were English-speaking male and female college students, aged 18-24 (*Mage* = 20.84). The majority of participants were female (n = 25) and the remainder were male. Most of the participants were White (n = 16), followed by African American (n = 8); the remaining participants were from a variety of ethno-racial identities.	Qualitative - Focus groups/interview. Each of the focus group discussions lasted 60–90min and was cofacilitated by two trained research assistants. The individual in-depth interviews lasted approximately 60min. The procedures implemented were consistent across the focus groups and individual interviews. At the beginning of each focus group/interview, participants were instructed to progress through the app prototype preloaded onto an iPod touch. Participants also had access to the app throughout the focus group/interview. The emphasis of the interviews was on the friend’s assessment of the app. The audio-taped interviews were digitally recorded and then transcribed.	Dating violence (DV)	MyPlan – a prototype smart phone application (app) that is a safety decision aid designed to assist college women (age 18–24) experiencing dating violence/survivors of dating violence and their friends who wish to learn more about how to help them. A collaborative, multistate research team partnered with the One Love Foundation, a national relationship violence prevention advocacy organization, to develop the app.	Three themes were directly related to participants’ perceptions of the benefit of MyPlan in helping themselves as well as their friends in addressing DV: usefulness, understandability, and appropriateness. The findings support the acceptability and usefulness of an app to support peers of DV survivors on campus and thereby also strengthen the safety net for DV survivors.
[10]	Enlisting friends to reduce sexual victimization risk: There's an app for that... but nobody uses it	United States	To collect feasibility and acceptability information on the Co6 app among college women who drink alcohol, a group at greater risk for sexualized violence, to shed light on the Co6 app and the challenges associated with app-based prevention in real-world contexts.	Forty-four college women. Women had to 18–24 years of age (*Mage* = 20.11, SD = 1.33), be enrolled in college, own a smart phone, and drink alcohol at least once per week in the last 6 months. A majority of participants were White (n = 23), followed by African American (n = 7), Asian (n = 6), Hispanic (n = 3), and other (n = 5). Almost all of the participants identified as heterosexual (n = 41)	Mixed Methods – Participants completed questionnaires, used the Co6 app for 2 months, and returned to report their experiences. For the qualitative component, participants were interviewed individually in a semi-structured format about what they liked and did not like about the app. Follow up interviews were approximately 1 hour long and were audio-recorded and transcribed verbatim.	Sexual victimization	Circle of Six (Co6 app) - centralizes both personal and community resources to reduce SV risk. Specifically, the app calls for users to program the con tact information of six trusted individuals, who are then identified as part of the user’s safety network (i.e., circle of six).	Findings were separated by what participants liked and what participants disliked. Themes related to what participants liked included that the app provided easy connection with friends, the app features, and believed the app was good in theory. Themes related to what participants disliked included that they thought the app was unnecessary, they were uncomfortable with group messaging, and there were limited contexts for use. Overall, the app may not meet the real-world needs of college women.
[11]	Developing an App for College Women in Abusive Same-Sex Relationships and Their Friends	United States	To establish initial content validity, feasibility, appropriateness, understandability, and usability of a smartphone-based safety decision aid app for college women in same-sex relationships and their friends.	Thirteen participants participated in interviews. These included eight college students (four female survivors, three female friends, and one male friend), five of whom were White and three African American, with a mean age 22.0 years, SD 1.9. Five college staff who worked directly with LGBT survivors on campus also participated (four female, one male; all White, mean age 28.2, SD 3.6) in the study.	Qualitative - Interviews using a semi-structured interview guide with questions regarding understandability, appropriateness, comprehensiveness, and usefulness of the app for women in same-sex relationships and their friends. Interviews were audio-recorded.	Same-sex dating violence	An interactive, personalized safety decision aid smartphone application (app) developed by a collaborative, multistate team. The app was intended to allow abused college-aged women and their friends to privately and safely assess violence severity in an abusive relationship, clarify their areas of decisional conflict, (e.g., advantages/disadvantages of the relation- ship) and identify their safety priorities and link to national resources (e.g., national hotline).	Findings focused on *barriers to recognizing abuse and accessing help* (three themes emerged: isolation, lack of awareness of abuse/violence and resources for support, and fear of or actual experiences of discrimination); and *feasibility of an app-based safety planning resource* (three themes emerged: appropriateness and inclusivity of app content for same-sex survivors and friends, appropriateness and acceptability of a smartphone-based approach for same-sex survivors and friends, and potential safety issues with the app). Overall, findings support the use of the app to assist college women experiencing same-sex dating violence and peers to connect with resources and develop tailored safety plans to reduce violence and increase their safety.
[32]	Usability testing of a mobile health intervention to address acute care needs after sexual assault	United States	To test the usability of a mobile health intervention targeting alcohol and drug misuse, suicide prevention, posttraumatic stress symptoms, coping skills, and referral to formal assistance for individuals who have experienced sexual assault.	Thirteen participants (*Mage* = 28.00) who experienced sexual assault and received a sexual assault medical forensic examination. Most participants identified as white (n = 13), female (n = 11), and were single (n = 7). One person identified as male and another as “other”. Approximately two-thirds of participants were not in college (*n* = 10) and had medical insurance (*n* = 10). The average length of time since the sexual assault was 12.09 months. The assaults were perpetrated by an acquaintance (*n* = 7), stranger (*n* = 5), and partner (*n* = 1).	Mixed Methods- The qualitative component consisted of individual interviews that were conducted in-person or through teleconferencing, according to participant preference, and lasted 45 to 60 minutes.	Sexual Assault	*SC-Safe* - a resource designed for individuals over the age of 18 residing in South Carolina who have experienced sexual assault. It was designed by the first and second authors to address a gap in clinical services after recent sexual assault.	Core themes included aesthetics and usability (app is simple and not overwhelming, layout allows for privacy, increase colour brightness and font size, make navigation functions clear and uniform across app); barriers to resources (logistical barriers, attitudinal barriers); and opinions about *SC-Safe* (education module was informative and helpful, feedback on emotion and behavioural health module, feedback on general coping skills). Overall, participants found the app to be user friendly and liked it more than they disliked it.
[44]	Survivor feedback on a safety decision aid smartphone application for college-age women in abusive relationships	United States	For young women who had previously experienced dating violence to evaluate a mobile phone application safety decision aid prototype, which was designed for use by college-age women experiencing dating violence.	Thirty-four English-speaking female college students, ages 18–25 (*Mage* = 21.26, SD = 1.86), who reported that they experienced dating violence while in college. Self-identified ethno-racial background were 52.6% White, 23.7% Hispanic, 7.9% African America, 13.2% Multiracial, and 2.6% other. Nearly 16% of participants reported being in a previous relationship with an abusive female partner.	Qualitative- Ten focus group (ranging from 2 to 7 participants) discussions, each lasting approximately 90 min, were cofacilitated by two trained research assistants in a campus or community location. Individual interviews took place in a setting of the participant’s choosing, were approximately 60 min, and were conducted by one trained research assistant. Procedures were consistent across the focus group sessions and individual interviews and a semi-structured interview guide was utilized.	Dating Violence/Intimate Partner Violence	A prototype smart phone application (app) that is a safety decision aid designed to assist college women (age 18–24) experiencing dating violence/survivors of dating violence and their friends who wish to learn more about how to help them. A collaborative, multistate research team partnered with the One Love Foundation, a national relationship violence prevention advocacy organization, to develop the app.	Participants reviewed and provided feedback on the app and four themes emerged: usefulness, understandability, appropriateness, and comprehensiveness of the app. Participants were positive about the potential of the app to provide personalized information about abusive dating relationships and appropriate resources in a private, safe, and nonjudgmental manner. Participants also provided recommendations for further development of the app.
Ragavan et al. [52]	Thrive: A Novel Health Education Mobile Application for Mothers Who Have Experienced Intimate Partner Violence	United States	Describe the development and formative evaluation of a trauma-informed, user-friendly Smartphone- based mobile application to address the unmet health needs and improve the well-being of mothers who have experienced intimate partner violence (IPV).	Eight IPV survivors and 16 hospital-based staff (nine health care providers, four social workers, one mental health provider, and three IPV advocates; hereafter called providers).	Formative Evaluation - Participants were instructed to use *Thrive* on a study Smartphone for 10 to 20 minutes and then provide feedback about its content, design, safety features, and applicability via a structured interview.	Intimate Partner Violence	Thrive - a trauma-informed, user-friendly Smartphone based mobile application (app) to address the unmet health needs and improve the well-being of mothers who have experienced IPV. A multidisciplinary team of IPV experts developed the app in partnership with software developers.	Participants found Thrive to be user-friendly, informative, trauma- informed, and easier and more relevant than other forms of health education. Participants had several recommendations including making the app more interactive and personalized by allowing users to add their own content, having a password to increase security, and providing social support mechanisms. Initial feedback sessions have demonstrated preliminary acceptability of the app.
Tarzia et al., 2017	“Technology Doesn’t Judge You”: Young Australian Women’s Views on Using the Internet and Smartphones to Address Intimate Partner Violence	Australia	To confirm the hypothesis that technology has a potential role in responding to IPV, and to ascertain what factors might encourage or discourage women from using an IPV website or app.	Nineteen women between 20 and 25 years of age. All participants were residing in Victoria, Australia at the time of the study, and all had self-reported experiencing fear of a partner in the previous 6 months. None of the women were married at the time of participation, and most were tertiary educated.	Qualitative- Four focus groups were held at The University of Melbourne between April and August 2014. The sessions were informal and semi structured in nature, and facilitated by a trained researcher. An additional note taker was present but did not take part in the conversation. The discussions lasted approximately 60 min each and were audio recorded and later transcribed verbatim by members of the research team.	Intimate Partner Violence	Not specified – general exploration of safety apps	Young women’s views around responding to IPV using web-based applications can be grouped into three main themes: behavioral beliefs and attitudes (it’s easier than telling someone, it’s not “normal” to be in an abusive relationship, an app can raise awareness, an app should do more than provide information, an app needs to strike a balance); normative beliefs and subjective norms (for young people technology is a way of life, it needs to be endorsed by someone who counts); and control beliefs and perceived behavioral control (access anywhere/anytime, protecting safety and privacy). Findings highlight the potential for technological interventions to become a valuable addition to the resources available to young women.

**Table 4:** Description of Safety Apps in Included Studies

Name	Founded By	Target Population	Purpose	Features	Connection to safety and sexualized violence
**MyPlan** [2, 44]	Collaborative, multistate research team (Arizona, Maryland, Missouri, and Oregon) partnered with One Love Foundation	College women (age 18-24)	Safety decision aid for those experiencing dating violence, survivors of dating violence, and their friends who wish to learn more about how to help	- Inconspicuous name and logo - Safety information (e.g., myths and reality about dating violence; possible “red flags” to look out for; abuse and safety concerns such as physical violence, reproductive control, how alcohol and drugs affect safety, stalking) - Password protection and automatic locking - Allows you to enter information about relationship, including severity of violence and safety priorities to provide a personalized risk assessment and safety plan - Provides national and local resources (e.g., hotlines with skilled advocates; professionals, college administrators, women’s centers, campus/local police, healthcare providers)	Safety decision aid for individuals experiencing **dating violence,** as well as a resource for their friends
**Circle of Six** [10]	Nancy Schwartzman (CEO), Thomas Cabus (Creative director), and Nick Hargreaves (Tech consultant) – created in 2015, winner of *Apps Against Abuse Challenge* by White House Office of Science and Technology	College women (age 18-24)	Quick communication for safety and support when in a situation where sexualized violence is occurring; sends pre-programmed group text messages to the user’s circle (trusted contacts)	- Programming of six trusted individuals who are part of the user’s safety network (the user’s “circle”; e.g., friends, family, co-workers) - Pre-programmed group text messages to user’s circle, including user’s GPS location, for immediate help/support - Internet links to health and safety resources, national hotlines, and “I am safe” text messages to the user’s circle	Safety of the individual when experiencing **sexual victimization** or to avoid experiencing **sexual violence**
**Unnamed app**, similar to MyPlan [11]	Collaborative multistate team (Oregon, Arizona, Missouri, and Maryland)	College aged women	Allow abused college-aged women and their friends to privately and safely assess violence severity in an abusive relationship by increasing their understanding of the situation to help with decision-making, clarify their areas of decisional conflict (e.g., advantages/ disadvantages of the relationship), identify safety priorities (e.g., privacy, feelings for partner, severity of violence and social support) and link to national resources (e.g., national hotline)	- Password-protected login - Homepage: introduction, identify if looking for information for own relationship or for a friend, and gender of the abusive partner - Healthy relationship information including: ○ warning signs of unsafe intimate same-sex relationships ○ dynamics of unsafe same-sex relationships ○ myths - Risk assessment tools (e.g., Danger Assessment and Danger Assessment-Revised to assess danger) - Priority-setting activity: “a series of pairwise comparisons of the relative importance of factors when making decisions about safety” - Safety plan with resources and information (e.g., national hotlines, trusted campus resources)	Safety decision aid for individuals experiencing **same-sex dating violence,** as well as a resource for their friends
**SC-Safe** [32]	Amanda K Gilmore and Tatiana M Davidson (first and second author of article)	Individuals aged 18+ in South Carolina having experienced sexual assault	Address a gap in clinical services after recent sexual assault, such as the inclusion of mental health services related to their experience of sexualized violence	- Screening and brief intervention using evidence-based practices to model post-sexualized violence services delivered in clinical setting - Intervention modules meant to inform and educate regarding various topics and their relation to sexualized violence: 1) alcohol and substance use, 2) suicide prevention, 3) posttraumatic stress and depressive symptoms, 4) adaptive coming skills, 5) physical health - Referral to treatment through community resources (e.g., testing for sexually transmitted infections, mental health interventions, support after sexualized violence, as well as links to local, regional, and national treatment settings and organizations) - Personalized psychoeducation and coping skills module through brief descriptive text and interactive learning exercises	Provide clinical services to individuals having experienced **sexual assault**, including information on have to stay safe and avoid or minimize chances of further sexualized violence
**Thrive** [52]	Multidisciplinary team of intimate partner violence experts (community-based IPV advocates and general pediatricians/IPV researchers) in partnership with software developers	Mothers who have experienced intimate partner violence	Trauma-informed health education app for intimate partner violence survivors and their children	- Information about local resources sensitive to intimate partner violence - Three education-based sections: *Myself* (maternal self-care, coping skills, and trauma-informed yoga); *My Child* (focused on childhood stress, promoting mother-child communication, and talking to children about IPV); *My Life* (resources about housing, education, childcare, IPV agencies, and national and state IPV and parenting hotlines)	Providing support, education, and information for mothers who are survivors of **intimate partner violence** and their children

### Narrative Summary of Themes

Using thematic analysis, three themes emerged that impacted women’s experiences of using sexualized violence safety apps: (1) security; (2) accessibility; and (3) knowledge. Recommendations and barriers found in relation to each theme are presented as subthemes. Table 5 offers a summary of each theme.

**Table 5:** Summary of the Themes

Themes	Specific Aspects	Sources	Sample Quotations
Security	Privacy, judgement, stigma	Blayney et al. [10]; Bloom et al. [11]; Gilmore et al. [32]; Lindsay et al. [44]; Ragavan et al. [52]; Tarzia et al. (2017)	*The option of ... being able to maybe correspond with people anonymously, especially if you’re scared of being judged or found out ... that’d be really good.* (Tarzia et al., 2017: p. 205).
Accessibility	Ease and usefulness	Alhusen et al. [2]; Blayney et al. [10]; Bloom et al. [11]; Gilmore et al. [32]; Lindsay et al. [44]; Ragavan et al. [52]; Tarzia et al. (2017)	*It helps you organize your mind because when you’re in the situation, you don’t really know how to feel ... there’s so much going on, you don’t really know how to categorize things. When [the app does] it for you, it just helps you put yourself in order, and have more control on your life. When something’s happening to you like that, you feel like you’re out of control and you can’t—you don’t know where to go. You don’t know what to do. You’re just so confused, so I think it helps.* ([44]; p. 378).
Knowledge	Information, awareness, validation, myth debunking	Alhusen et al. [2]; Bloom et al. [11]; Gilmore et al. [32]; Lindsay et al. [44]; Ragavan et al. [52]; Tarzia et al. (2017)	*I liked the concrete advice...it didn’t just say “talk to your friend”— rather “here are five different things you can say.” I feel like everyone knows you have to talk to the person but people don’t know what to say and how to say it, and that was very, very useful.* ([2]; p. 274).

### Security

Security was found to be a prevalent theme across studies that influenced women’s experiences of using safety apps [2, 10, 11, 32, 44, 52, 64]. Specific aspects of security that were discussed included privacy, judgement, and stigma. All of the studies identified that using safety apps to obtain information provided more privacy or anonymity than obtaining information from in-person health services [2, 10, 11, 32, 44, 52, 64]. Using a safety app was also found to provide additional privacy because individuals could access them discreetly and apps were designed to be ambiguous to other; for example, using basic colours that would not be recognized as a sexualized violence related app by another person who might see the individual’s phone [32, 52, 64].

Four studies noted that participants experienced a greater sense of security because they faced less judgement regarding being a victim of sexualized violence and, therefore, avoided stigma when using a safety app [11, 32, 44, 64]. Using a safety app was found to be more objective and unbiased than accessing traditional health services or speaking with friends and family [32, 44, 64], and five of the seven studies acknowledged that individuals found it was easier to interact with an app than having to discuss their situation with health professionals or their social network [2, 11, 32, 44, 64]. Women perceived less stigma about having experienced sexualized violence as a result of using these apps [11, 32, 44, 64]. For example, one participant identified:

With suicide already being stigmatized the way it is, and communication about suicide being the way it is, I would want to know that like, it’s okay to talk about this and it’s okay if this is what you’re feeling like ([32], p. 10).

Many participants mentioned the option of not having to discuss sexualized violence in a traditional way such as “face-to-face” as a benefit [2, 11, 32, 44, 64]. One participant noted, “it [app] gives you a privacy and accessibility . . . the fact that I don’t have to go to Student Health Center to get help and not have to worry about being judged” ([2], p. 276). Another participant discussed the benefit of accessing help via an app instead of going to a counseling center on campus, noting: “I feel judged to go . . . just knowing that I’m going to see them every day since I live there [on campus] I would feel a little uncomfortable” ([2], p. 276). Interestingly, several studies identified that it would be beneficial to be able to engage with others through the app [44, 52, 64], stating that “the option of ... being able to maybe correspond with people anonymously, especially if you’re scared of being judged or found out ... that’d be really good” ([64], p. 205).

#### Barriers and recommendations

The greatest barrier that emerged in relation to security was privacy, including the potential for partner monitoring or surveillance which may limit use of the app [11, 32, 44, 64]. For example, one participant commented: “If somebody’s in a relationship that is abusive, and someone’s already checking their phone and checking everything they’re doing, and they have an app about this on their phone, it might cause issues” ([44], p. 382). Recommendations to address privacy concerns, some of which were already part of the apps studied, included providing password protection for the app [2, 44, 52, 64], an emergency exit on each screen [11, 52], use of an innocuous name for the app that does not refer to relationships or safety [2, 11, 44, 52, 64], and even allowing the user to rename the program or change the icon themselves [11].

Bloom et al. [11] suggested the ability for users to print or e-mail the contents of the app to themselves or another person and then delete the app or the answers as a useful alternative. This would circumvent the need for the resources on the app to be exclusively on a computer or smartphone [11]. Other studies specifically recommended educating users about healthy relationship boundaries regarding technology and sharing passwords with partners and friends to better instruct users how to hide the app (e.g., bury the app in smartphone folders), and how to safely use the app if a partner monitors their phone [11, 44]. Women who reviewed the Circle of 6 (Co6) app specifically identified being uncomfortable with the group messaging feature, which is customized to send messages to only select individuals that you identify as your “circle of 6” [10].

### Accessibility

The importance of accessibility emerged as a key theme that influenced experiences that women had when using sexualized violence safety apps. Accessibility included the ease and usefulness of the safety apps [2, 10, 11, 32, 44, 52, 64]. Women in all studies reported that they found the safety apps to be user friendly, easy to use, and easily accessible [2, 10, 11, 32, 44, 52, 64]. In six studies, the usefulness of the app was directly related to the fact that it could be used anywhere, was comprehensive in content, and all the information needed was in one place [2, 11, 32, 44, 52, 64]. For example, one participant commented:

It helps you organize your mind because when you’re in the situation, you don’t really know how to feel ... there’s so much going on, you don’t really know how to categorize things. When [the app does] it for you, it just helps you put yourself in order, and have more control on your life. When something’s happening to you like that, you feel like you’re out of control and you can’t—you don’t know where to go. You don’t know what to do. You’re just so confused, so I think it helps ([44], p. 378).

In many studies, participants found that the app could be customized or personalized to the specific user, which improved its accessibility and overall usability [2, 10, 44]. Examples of personalized or customized content included safety planning suggestions [2], messages that will be sent to friends [10], and what information is presented, such as a specific risk assessment and safety plan for the user [44]. Overall, most studies found that the accessibility of safety apps had the potential to improve safety and decrease risk [10, 11, 32, 44, 52, 64].

#### Barriers and recommendations

Several studies noted suggestions to improve the accessibility of sexualized violence safety apps. Making the apps more personalized and interactive was recommended by women in five studies [10, 11, 44, 52, 64]. Examples included providing written scripts to choose from [10, 11, 32] or the ability to connect directly with a counsellor through the app [44, 52, 64]. Other recommendations included making the navigation functions standard across the app and clearly indicating the purpose of each icon [32]. Celebrity endorsement was also suggested as a way to promote sexualized violence safety apps, which in turn would increase a sense of accessibility for more women [64].

Alhusen et al. [2] noted that if individuals are not ready to address the violence, then the app may be unnecessary and useless. One participant noted “If they’re not ready they’re not ready… don’t talk about them behind their back and don’t talk about them with others [on an app] ([2], p. 276)”. Further, women in the Blayney et al. [10] study noted the app had limited contexts for use and did not provide anything more than a mobile phone could offer, seeing the app as unnecessary. Feedback included:

It just generally seemed like you could do the same things without the app, because iPhones nowadays are so intricate. Like, you could click details on your messages and press ‘send location’ and type a short message. I feel like that wouldn’t take nearly as long as opening the app, clicking the button, sending the messages… It’s not really an easy way to contact friends, I think personally for me, it would just be easier to call or text them. Like it wasn’t any easier to do that [use the app] ([10], p. 771).

### Knowledge

Six studies discussed the importance of knowledge in the experiences of women when using sexualized violence safety apps [2, 11, 32, 44, 52, 64]. How women experienced the knowledge provided on the safety apps was dependent on the information provided, and if the information increased awareness, validation, and myth debunking. In all six studies, participants found the safety apps to be helpful in raising awareness of sexualized violence and recognizing violent behaviour through various ways such as myth debunking [2, 11, 32, 44, 52, 64]. Additionally, six studies found the apps to be an easy way to acquire information that validated women’s experiences of violence and indicated that participants found the app provided assurance that they are not alone [10, 11, 32, 44, 52, 64].

Further, six studies found that women believed the apps to contain relevant information that was credible, evidence based, and/or trauma informed [2, 11, 32, 44, 52, 64]. In relation to providing knowledgeable and credible information, one participant commented:

I liked the concrete advice...it didn’t just say “talk to your friend”— rather “here are five different things you can say.” I feel like everyone knows you have to talk to the person, but people don’t know what to say and how to say it ([2], p. 274).

Similarly, another participant commented that the app “arms you with ideas as to how to go about it [conversations] properly” ([2]; p. 276).

The information, options, choices, and safety planning strategies provided on the safety apps were also found to increase a feeling of empowerment [2, 11, 44, 64]. For example, one participant commented: “[A young woman] should feel relieved. Like she is equipped to know what to do, and not lost and drowning her sorrows and burden by herself. Like someone is there to help her” ([64] p. 209).

#### Barriers and recommendations

Several studies noted recommendations for improving the knowledge provided on sexualized violence safety apps. Several women thought that personal anecdotes, rather than statistics about dating violence, might better help young women recognize the violence in their own relationships [11, 52]. Gilmore et al. [32] and Lindsay et al. [44] found that some young women noted the desire for more information throughout the app about emotional abuse, as illustrated by the following participant: “It would be really cool if there was more stuff about emotional abuse and control because I think that is also really important” ([44], p. 383). Survivors thought information about what the police can and cannot do to assist the survivor would also be helpful because “talking to police can be kinda scary” ([44], p. 383). Last, several studies believed that sexualized violence safety apps should do more than just provide information [11, 32, 44, 64]. In addition to providing information, the safety apps need to expand the ability to gain knowledge by including information for appropriate resources [11, 32, 64] and incorporating further educational modules and learning opportunities [11, 32].

## Discussion

This scoping review was conducted to explore the nature and extent of literature on women’s experiences of safety apps related to sexualized violence. To our knowledge, this is the first scoping review to explore this topic. While most safety apps are studied through quantitative measures to determine prevalence of use and downloads, as well as content creation (e.g., [30, 46]), quantitative data is limited in its ability to capture the lived experiences of participants [29]. The current scoping review therefore was designed to focus solely on qualitative studies. Within the literature reviewed, three common themes emerged that influenced women’s experiences of sexualized violence safety apps: security, accessibility, and knowledge. This review, however, confirms that the qualitative literature on women’s experiences of sexualized violence safety apps is scarce and exposes a gap in the literature on this topic does indeed exist.

Of the seven studies included, the majority of the samples comprised of Caucasian/White females, and there was an under-representation of other ethno-racial groups [2, 10, 11, 32, 44]. Moreover, only one study disclosed the sexual orientation of their participants, which comprised of predominantly heterosexual females [10]. There was one other study that included a participant that identified as neither female nor male [32], which is insufficient to capture the experiences of gender-non-conforming and trans persons. Given the widespread evidence that members from diverse ethno-racial identities and the sexual and gender diverse community are often victims of unique forms of sexualized violence [23, 38, 43], our review has highlighted a significant gap in this literature. Future research should aim to recruit samples that are entirely, or predominantly, comprised of members of diverse communities to better understand their unique experiences and needs regarding sexualized violence safety-apps. There is a need for research that explores the unique experiences of women with diverse gender and ethno-racial identities.

From the included studies in this review, it became evident that feelings of security and privacy were of utmost importance to the women using the apps [10, 11, 32, 44, 52, 64]. Some women felt an increased sense of privacy while using the app, which allowed them to feel more comfortable and secure, as well as less judged and stigmatized by others. While certain apps were praised for their discrete icons and layouts [11, 32], one article specifically discussed the for improvements in this area through increased password protection and a “quick exit” button or feature to disguise the app’s purpose to an onlooker [52]. The concept of privacy on apps has become a key concern for many app users, often in regards to sharing of sensitive user data [6, 28], or where data will be stored [35]. For health apps specifically, a fear surrounding privacy lies in the potential of unauthorised use or disclosure of health information, which could lead to social stigma and discrimination [39]. A quantitative study with a focus on how privacy is valued by app users across a variety of apps (e.g., care sharing, diabetes app, companion and security app, and mood adjustment app) found that premium (i.e. at a cost) privacy features were more favourable than others, such as premium functions and personalization [27]. As demonstrated through the literature, most privacy concerns were related to sharing personal information, whereas the studies in this review discussed privacy in the context of shielding their use of the app from a partner or from social networks.

Our review found that accessibility, inclusive of how easy and useful the app was perceived to be, was integral to the user experience. App content and design has been directly related to accessibility and the rate of use in other literature. The connection between ease of use and a user’s experience has been widely discussed, especially among health apps (e.g., [4, 26, 55, 72]). For example, a qualitative study exploring user experiences of mobile health apps found that an apps ease of use led to more desire to use the app [4]. Features that made the app easy to use were automation of in-app functions that reduced time in performing tasks, and convenience such as having information for a person’s self-management plan in one location [4]. In a qualitative study on barriers and facilitators of medical mobile app use, app features such as information content, accessibility of the information, and ease of use of the app, were highlighted as facilitators that promoted use of the app [72]. Another study, which explored factors that influence use of a mobile app for reporting adverse drug reactions and receiving safety information, found that use of the app was influenced by ease of use and the security of the app [26]. Overall, it is apparent that an apps ease of use is an integral part of the user experience.

The reviewed literature found that increased information, awareness, validation, and myth debunking were positive knowledge features on sexualized violence safety apps [2, 11, 32, 44, 52, 64]. It was also found that users appreciated information that was trauma informed, credible and evidence based [2, 11, 32, 44, 52, 64]. Similar to our findings, a study examining help-seeking for domestic violence victims found that apps were a particularly good avenue for accessing information [25]. Other literature has reported that mobile apps with high quality education materials can significantly increase the knowledge of users [40] and directly influence their attitudes and behaviours [42, 58]. It is important to note that sexualized violence safety apps can also be used to inform individuals who are not victims or perpetrators, such as bystanders. Shaw and Janulis [57] found that bystander education increased sexualized violence knowledge, decreased the likelihood of endorsing rape myths, and increased a sense of efficacy for intervening as a bystander. Last, apps provide a convenient and affordable way to access information that is often interactive and are an environmentally friendly alternative to information that is often conveyed on paper [42].

The use of technology to increase safety is not a novel concept. For example, ride-share apps such as Uber offer GPS tracking that can be shared with family and friends in real time, as well as a distress alarm available on the app that can signal the ride-sharing service for help [21]. Increased safety through technology can also be in the form of delivering safety information, such as the use of mobile technology for delivery safety awareness in the workplace [54] and communicating emergency safety information through mobile text alerts [73]. Safety in relation to technology and sexualized violence, however, has primarily been discussed negatively. The use of social media, personal tracking applications, and smartphone technology in general have been found to facilitate forms of sexualized violence, such as harassment, stalking, violence, and dating abuse [38]. Interestingly, despite these challenges, a study by Finn and Atkinson [30] found that women who experience sexualized violence through technology still feel a sense of independence when using technology. This aligns with the findings presented in this review, which found that the women who had experienced sexualized violence felt that safety apps provided them with increased knowledge, anonymity, validation, and a sense of empowerment [11, 44, 64].

### Limitations

Quality appraisal is not a compulsory component of the Arksey and O’Malley [5] scoping review framework, and the included studies in this scoping review were not critically appraised. Although this scoping review did not seek to assess quality of evidence, it consequently cannot determine whether the included studies provide robust or generalizable findings or if the research itself is of poor quality. Literature that was not included in this review included reviews, commentaries, editorials, and conference proceedings. Existing networks and relevant organizations were also not contacted. While the search was broad, some relevant studies may have been omitted. In addition, the review only included literature published in English and French and, therefore, relevant literature may have been omitted if published in other languages. Although many apps are currently being created and developed, they may not be at the user testing phase or are not explicitly looking at users’ experiences beyond interface evaluations. Despite these limitations, the review does provide important understandings of women’s experiences of sexualized violence safety-apps.

## Conclusion

This scoping review provides a comprehensive summary of the qualitative research findings in relation to women’s experiences of sexualized violence safety apps. This review has highlighted that there is limited research conducted in this area. Victims of sexualized violence are in a unique position to provide insight to app developers on their priorities and specific needs, which can potentially change the way women utilize app technology for their safety. Additional research that focuses on the experiences of women users will help to better inform quality app development that is secure, informative, useful, and wanted by the user. Overall, the reviewed literature in this study found safety apps to be a private all-in-one resource for support, information, and emergency planning that were useful and easy to use. Further, women believed that sexualized violence safety apps had the potential to decrease the overall risk of experiencing sexualized violence.

## Supplementary Information

**Additional file 1.**

## Declarations

### Availability of data and materials

N/A
